# Obesity and bone health: reconciling the density–fragility paradox

**DOI:** 10.3389/fendo.2026.1864169

**Published:** 2026-07-10

**Authors:** Riad Sulimani

**Affiliations:** Department of Medicine, College of Medicine, King Saud University, Riyadh, Saudi Arabia

**Keywords:** inflammation, obesity, osteoporosis, oxidative stress, sarcopenic adiposity

## Abstract

The relationship between obesity and bone health is increasingly recognized as complex and paradoxical. Despite higher bone mineral density (BMD) in individuals with obesity, fracture risk is not uniformly reduced. Instead, fractures tend to occur more frequently at peripheral sites such as the ankle, humerus, and tibia, indicating a mismatch between BMD and true bone strength. In the early stages of weight gain, mechanical loading and adipose-derived hormonal factors may promote bone formation. However, these effects are progressively counterbalanced by chronic inflammation, oxidative stress, and marrow adiposity, which impair bone quality and compromise microarchitecture. Conventional dual-energy X-ray absorptiometry (DXA), while central to clinical assessment, cannot capture these changes and may overestimate skeletal strength in the presence of excess soft tissue. Emerging tools such as trabecular bone score and high-resolution peripheral quantitative computed tomography provide additional insights into bone quality, including microarchitecture and cortical porosity, particularly in individuals with type 2 diabetes. Obesity is also associated with vitamin D deficiency, impaired muscle function, and sarcopenic phenotypes, further increasing fracture risk. Bariatric surgery introduces additional challenges by accelerating bone turnover and loss. This review highlights the need to move beyond BMD alone and adopt comprehensive, individualized approaches to fracture risk assessment and management in obesity.

## Introduction

Obesity and osteoporosis are major global health challenges. The prevalence of obesity has risen substantially over recent decades (1), while osteoporosis remains a significant cause of morbidity and fracture worldwide (2). Although individuals with higher body weight often demonstrate greater bone mineral density (BMD) at weight-bearing skeletal sites, accumulating evidence indicates that this does not reliably translate into stronger bone or lower fracture risk. Large cohort studies show that obesity is associated with a distinct fracture pattern—lower risk at the hip and spine but higher risk of fractures at peripheral sites such as the ankle, humerus, and tibia (3–5). These observations highlight a dissociation between bone density and bone quality that is particularly relevant for clinical densitometry. Dual-energy X-ray absorptiometry (DXA) may overestimate skeletal strength in individuals with obesity due to soft-tissue interference and the inherent limitations of two-dimensional, areal rather than volumetric measurement (6). As a result, skeletal fragility may remain undetected unless complementary tools are used. Advances such as the trabecular bone score (TBS) and high-resolution peripheral quantitative computed tomography (HR-pQCT) offer improved assessment of trabecular microarchitecture, cortical integrity, and estimated bone strength—particularly valuable in individuals with metabolic dysfunction or type 2 diabetes (7–10). The skeletal phenotype associated with obesity reflects the combined influence of mechanical loading, endocrine factors, chronic low-grade inflammation, oxidative stress, and expansion of marrow fat (11–13). Additional contributors—including vitamin D deficiency, reduced muscle strength, sarcopenia, and bone loss after bariatric surgery—further compromise structural resilience and increase the risk of falls and fractures (14–17). Understanding how these mechanisms interact is essential for accurate diagnosis and individualized management. This review summarizes the pathways linking excess adiposity to skeletal fragility and outlines practical strategies for clinical evaluation and treatment.

## Positive effects of obesity on bone

### Mechanical loading and skeletal adaptation

Increased body mass enhances mechanical loading, stimulating adaptive changes in bone through the mechanostat process. Repetitive loading promotes periosteal expansion, increased cortical thickness, and improved structural geometry at weight-bearing sites (18, 19). Osteocytes act as key mechanosensors; deformation of the lacuno-canalicular network activates mechanosensitive ion channels, integrins, and downstream MAPK/ERK and Wnt/β-catenin signaling pathways, promoting osteoblastogenesis and matrix mineralization (20–22). Mechanical strain also suppresses sclerostin production, further enhancing bone formation (23). The recently characterized Piezo1 ion channel offers an additional mechanistic link between mechanical stress and osteogenic gene expression (24). These adaptive responses may increase bone mineral density in mild to moderate obesity, although mechanosensitivity tends to plateau at higher levels of metabolic dysfunction (25).

### Endocrine and metabolic influences

Adipose tissue exerts endocrine effects that may temporarily support bone formation. Insulin enhances osteoblast activity via stimulation of collagen synthesis and osteocalcin production (26). Peripheral conversion of androgens to oestrogen through aromatase reduces osteoclastic resorption and helps preserve trabecular mass, particularly in postmenopausal women (27). Leptin stimulates osteoblastogenesis and suppresses osteoclast formation peripherally, although central sympathetic activation may offset some of these benefits (28, 29). Adiponectin demonstrates context-dependent skeletal effects, with experimental evidence showing both anabolic and catabolic influences depending on metabolic state (30, 31). Overall, endocrine and metabolic factors may enhance bone formation in early obesity, but these effects diminish as inflammation and insulin resistance emerge.

## Negative effects of obesity on bone

### Inflammation and cytokine signaling

Chronic low-grade inflammation is a hallmark of obesity and a major driver of skeletal deterioration. Visceral adipose tissue becomes infiltrated with pro-inflammatory macrophages that secrete cytokines such as tumor necrosis factor-α, interleukin-1β, and interleukin-6 (32, 33). These mediators stimulate osteoclastogenesis through up-regulation of the receptor activator of nuclear factor κB ligand (RANKL) pathway while simultaneously suppressing osteoblast differentiation and survival (33). The net effect is an uncoupling of bone remodeling in favor of resorption. Population-based studies consistently demonstrate that elevated inflammatory markers correlate with increased fracture risk, particularly among older adults with central obesity (33).

### Oxidative stress and impairment of osteogenic pathways

Obesity is associated with heightened oxidative stress, driven by lipid peroxidation, mitochondrial overload, and impaired antioxidant defenses (34, 35). Excess reactive oxygen species reduce osteoblast viability, disrupt osteocytic signaling, and enhance osteoclast activity (34, 35). Oxidative stress also suppresses Wnt/β-catenin signaling, a key regulator of osteogenesis, thereby shifting mesenchymal stem-cell differentiation toward adipogenesis at the expense of osteoblast formation (35). Accumulation of advanced glycation end-products within collagen further increases non-enzymatic cross-linking, leading to greater bone brittleness and reduced toughness (36).

### Marrow adiposity and structural fragility

Expansion of bone marrow adipose tissue is a defining feature of obesity-related bone fragility. Increased differentiation of mesenchymal stem cells into adipocytes reduces the pool of osteoprogenitor cells, resulting in diminished bone formation (37). Marrow adipocytes also secrete inflammatory adipokines and free fatty acids that locally impair osteoblast activity and promote osteoclastogenesis (38). Imaging studies using HR-pQCT in individuals with type 2 diabetes and obesity demonstrate increased cortical porosity, reduced trabecular number, and impaired trabecular connectivity (38, 39). Transcriptional regulators such as PRDM16 further influence mesenchymal lineage allocation and may contribute to the preferential expansion of marrow adiposity observed in obesity (40).

### Vitamin D deficiency and secondary hyperparathyroidism

Vitamin D deficiency is highly prevalent among individuals with obesity and contributes significantly to impaired skeletal health (14). Several mechanisms underlie this association, including sequestration of vitamin D within expanded adipose stores, reduced cutaneous synthesis due to limited sunlight exposure, and volumetric dilution related to higher body mass (14, 17, 41). Persistent vitamin D deficiency leads to secondary hyperparathyroidism, with elevated parathyroid hormone (PTH) levels increasing cortical bone resorption in order to maintain calcium homeostasis (17, 42). Over time, this adaptive response results in greater cortical porosity and reduced mechanical competence (42). Consequently, serum 25-hydroxyvitamin D concentrations are frequently low despite adequate intake or sun exposure (14, 17). Clinical guidelines generally recommend vitamin D supplementation of 1,000–4,000 IU/day to achieve and maintain sufficiency, although higher or more prolonged dosing may be required in individuals with obesity due to sequestration and dilution effects (42). Adequate calcium intake—typically 1,000–1,200 mg/day—is also necessary to support optimal mineralization (43).

In post–bariatric surgery patients, vitamin D and calcium management becomes more complex. Reduced gastric acidity and impaired intestinal absorption favor the use of calcium citrate rather than calcium carbonate (44). Long-term vitamin D deficiency is common after Roux-en-Y gastric bypass and sleeve gastrectomy, and regular biochemical monitoring—including serum 25(OH)D and PTH—is essential to prevent persistent hyperparathyroidism and accelerated bone loss (15).

Beyond skeletal effects, correction of vitamin D deficiency also improves muscle function and reduces falls. Meta-analytic data indicate that vitamin D repletion leads to a modest but clinically meaningful reduction in fall risk, which is particularly relevant in individuals with obesity who already exhibit impaired balance and reduced muscle quality (45). **Table 1** summarizes the positive and negative effects of obesity on bone.

**Table 1:** Positive vs negative effects of obesity on bone.

Aspect	Positive Effects	Negative Effects
Bone Mineral Density	↑areal BMD at load-bearing sites	DXA overestimates; fractures persist
Architecture	↑ Periosteal expansion; ↑ cortical thickness	Marrow fat → porosity & fragility
Mechanotransduction	↓ Sclerostin, ↑ Wnt signaling	Inflammation/ROS blunt response
Hormones	Insulin, IGF-1, leptin (peripheral), oestrogen	Central leptin, low adiponectin, inflammatory adipokines
Inflammation	–	Chronic low-grade inflammation (TNF-α, IL-6)
Oxidative Stress	–	↑ ROS → collagen cross-links, brittleness
Fracture Risk	↓ Vertebral risk/hip in some cohorts	↑ Peripheral fractures (ankle, humerus)
Muscle Interaction	↑ passive loading	Sarcopenic obesity → falls, fragility
Special Contexts	Aromatase-derived estrogen post menopause	T2DM, vitamin D deficiency, bariatric surgery

Obesity exerts both positive and negative influences on the skeleton. Mechanical loading, periosteal expansion, and hormonal factors (insulin, IGF-1, leptin, aromatized estrogen) tend to increase bone mass, whereas inflammation, oxidative stress, advanced glycation end-products, marrow adiposity, and increased cortical porosity compromise bone quality. The net effect is a phenotype characterized by high BMD but impaired microarchitecture (“dense yet fragile”). Diagnostic challenges include DXA overestimation, the need for TBS and HR-pQCT to assess microstructure, and limitations of FRAX in obesity. Therapeutic strategies incorporate lifestyle measures (resistance training, adequate protein intake) and pharmacologic options tailored to the patient’s fracture-risk phenotype.

## Sarcopenic and osteosarcopenic obesity

Sarcopenic obesity refers to the coexistence of excess adiposity with reduced skeletal muscle mass or strength, while osteosarcopenic obesity represents the combined presence of obesity, sarcopenia, and osteoporosis (46, 47). These phenotypes share common mechanistic pathways, including chronic inflammation, oxidative stress, mitochondrial dysfunction, and endocrine dysregulation (48–49).

### Pathophysiology in sarcopenic obesity

Visceral adiposity plays a central role in driving adverse musculoskeletal changes. Increased infiltration of adipose tissue by macrophages leads to secretion of TNF-α and IL-6, which impair myocyte protein synthesis and promote osteoclast-mediated bone resorption (33). Mitochondrial dysfunction and excess oxidative stress further diminish muscle strength and impair osteoblast function (13, 48). Declines in anabolic hormones such as growth hormone and IGF-1, combined with altered leptin and adiponectin signaling, contribute to progressive losses of both muscle and bone mass (28–30).

From a mechanical perspective, although higher body mass increases passive skeletal loading, reduced muscle strength limits active mechanical strain—an essential stimulus for bone adaptation. This imbalance explains why individuals with sarcopenic obesity may show preserved or elevated BMD yet remain highly susceptible to falls and fragility fractures (49).

Management requires an integrated approach targeting both muscle and bone. Adequate dietary protein intake (typically 1.0–1.2 g/kg/day) supports muscle protein synthesis and optimizes bone turnover (50, 51). Anti-inflammatory and antioxidant-rich dietary patterns may mitigate oxidative stress and improve musculoskeletal outcomes (51). Gradual rather than rapid weight reduction helps preserve lean mass and maintains skeletal loading (52).

Resistance training is one of the most effective therapeutic interventions, consistently improving muscle strength, lean mass, physical performance, and mechanical bone loading (53). The combination of resistance exercise with protein supplementation produces synergistic benefits for muscle mass and function (54).

Pharmacologic therapies, although adjunctive, may also contribute. Osteoanabolic agents such as teriparatide and romosozumab improve trabecular microarchitecture and may exert secondary benefits on muscle–bone crosstalk (55, 56). Emerging therapies—including selective androgen receptor modulators and myostatin inhibitors—are being actively investigated for their ability to deliver coordinated anabolic effects on both muscle and bone (57, 58).

## Diagnostic considerations in obesity-related bone disease

Interpretation of skeletal health in individuals with obesity presents important diagnostic challenges. Dual-energy X-ray absorptiometry (DXA), the standard tool for measuring areal bone mineral density, frequently overestimates true skeletal strength in obesity due to soft-tissue interference, increased body size, beam hardening, and the inherent limitations of two-dimensional measurement (6, 59, 60). As a result, individuals with obesity may appear to have normal or even elevated BMD despite the presence of significant deficits in cortical and trabecular microarchitecture (61, 62). This limitation is particularly relevant in patients with central adiposity and type 2 diabetes, where standard DXA-based thresholds often fail to accurately reflect fracture susceptibility (39, 63–65).

To overcome these shortcomings, incorporation of bone-quality measures has become increasingly important in clinical assessment (63, 64). The trabecular bone score, derived from lumbar spine DXA images, provides an indirect index of trabecular microarchitecture (65, 66). Lower trabecular bone score values are consistently observed in individuals with type 2 diabetes and central obesity and are independently associated with increased risk of major osteoporotic and hip fractures (65, 66). Importantly, trabecular bone score captures microarchitectural deterioration that may remain undetected by areal BMD alone, particularly in patients whose skeletal loading artificially elevates density measurements (9, 63).

High-resolution peripheral quantitative computed tomography offers additional three-dimensional assessment of trabecular number, cortical thickness, cortical porosity, and estimated biomechanical competence (9, 68). Although not yet widely available in routine clinical practice, this technology is particularly informative in metabolically unhealthy obesity, where it reveals cortical thinning, increased porosity, and deterioration of trabecular connectivity despite preserved BMD (67, 68). Together, these diagnostic tools demonstrate that reliance on areal BMD alone is insufficient for accurate skeletal risk stratification in obesity and underscore the need for integrated structural assessment whenever available (6, 9, 68).

## Fracture-risk assessment

Standard fracture-risk prediction models frequently underestimate fracture probability in individuals with obesity and in those with type 2 diabetes (69–71). This underestimation reflects the inability of conventional algorithms to fully capture altered bone material properties, increased cortical porosity, compromised microarchitecture, higher fall propensity, and the adverse skeletal effects of chronic hyperglycemia (67, 69, 70). As a consequence, many high-risk patients are incorrectly classified as low or intermediate risk when standard tools are applied without adjustment.

Incorporation of trabecular bone score into FRAX significantly improves fracture prediction in individuals with obesity and diabetes by integrating microarchitectural information into probability estimates (9, 66). When trabecular bone score is unavailable, validated practical adjustments can be applied to improve risk estimation, including downward adjustment of the femoral-neck T-score input by approximately 0.5 standard deviations, selection of rheumatoid arthritis as a surrogate risk factor, or increasing the patient’s age by 10 years to account for diabetes-related skeletal fragility (72).

In regions where FRAX has been recalibrated using local epidemiological data and population-specific risk coefficients, fracture risk prediction can be further improved (73–76).

Given the multidimensional nature of obesity-related skeletal fragility, fracture-risk assessment is most informative when these domains are considered together rather than in isolation. An integrated evaluation that combines areal BMD, trabecular bone score (when available), indices of metabolic health, cortical structure, muscle strength and fall risk, and prior fracture history provides a more realistic estimate of skeletal vulnerability (77–80).

## Future directions

Advances in artificial intelligence and machine learning are transforming fracture-risk prediction. AI-enhanced models incorporating TBS, HR-pQCT microarchitecture, and large-scale clinical data outperform conventional risk algorithms in identifying high-risk individuals (81–83).

At the therapeutic frontier, mesenchymal stem-cell–derived exosomes and metabolic reprogramming strategies show promise in restoring osteogenic potential in metabolically impaired bone (84, 85). Endocrine restoration strategies, including correction of hypogonadism, may help counter sex-steroid–deficiency–related increases in marrow adiposity, while emerging anabolic agents such as selective androgen receptor modulators and myostatin inhibitors may enhance lean mass and musculoskeletal function (86, 87).

**Figure 1:**
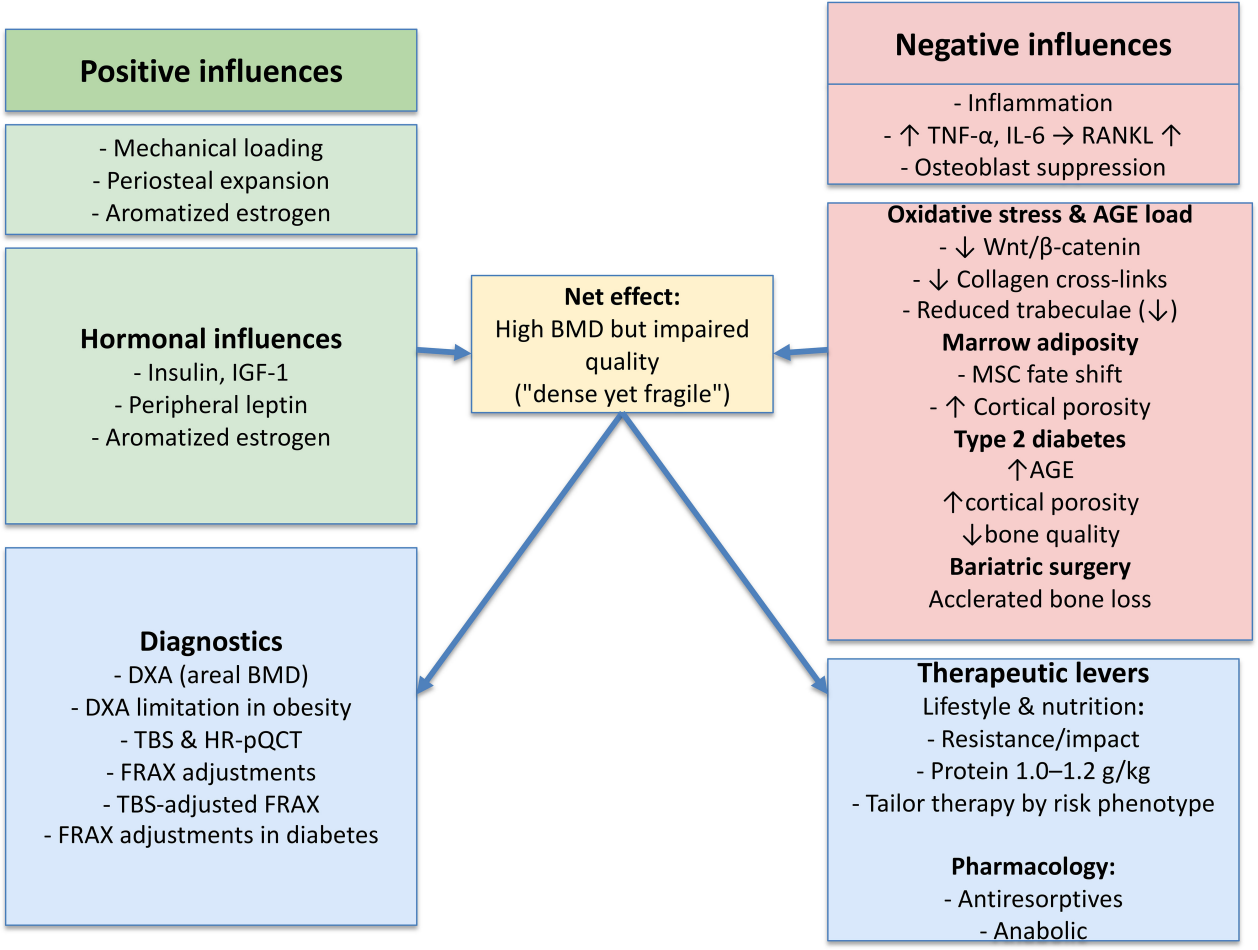
Mechanistic, diagnostic, and therapeutic framework for bone health in obesity.

The integrated framework is summarized in **Figure 1**, which links obesity-related positive and negative skeletal influences with diagnostic considerations and therapeutic levers.

## Conclusion

In summary, obesity creates a dense-yet-fragile skeletal phenotype in which higher areal BMD can coexist with impaired microarchitecture, reduced muscle quality, and increased peripheral fracture risk. Assessment should therefore move beyond BMD alone and integrate bone-quality measures, metabolic status, muscle function, fall risk, and fracture history. Management should be individualized and combine lifestyle, nutritional, and pharmacologic strategies according to the patient’s risk phenotype.

## References

[1] Ng M Fleming T Robinson M Thomson B Graetz N Margono C . Global prevalence of overweight and obesity. The Lancet. (2014) 384:766–781. doi: 10.1016/S0140-6736(14)60460-8 24880830 PMC4624264

[2] International Osteoporosis Foundation . Osteoporosis: A disease that affects the whole body. Nyon, Switzerland: International Osteoporosis Foundation (2021).

[3] Compston JE Watts NB Chapurlat R Cooper C Boonen S Greenspan S . Obesity and fracture risk in adults: results from the GLOW study. The American Journal of Medicine. (2011) 124:1043–1050. doi: 10.1016/j.amjmed.2011.06.013 22017783 PMC4897773

[4] Premaor MO Pilbrow L Tonkin C Parker RA Compston J . Obesity and fractures in postmenopausal women. Journal of Bone and Mineral Research. (2010) 25:292–297. doi: 10.1359/jbmr.091004 19821769

[5] Prieto-Alhambra D Premaor MO Fina Avilés F Hermosilla E Martinez-Laguna D Carbonell-Abella C . The association between fracture and obesity is site-dependent. Journal of Bone and Mineral Research. (2012) 27:294–300. doi: 10.1002/jbmr.1466 22095911

[6] Shepherd JA Schousboe JT Broy SB Engelke K Leslie WD . Executive summary of the 2013 ISCD Position Development Conference on DXA. Journal of Clinical Densitometry. (2013) 16(4):455–466. doi: 10.1016/j.jocd.2013.08.004 24183638

[7] Hans D Goertzen AL Krieg MA Leslie WD . Bone microarchitecture assessed by TBS predicts osteoporotic fractures independent of bone density: the Manitoba study. Journal of Bone and Mineral Research. (2011) 26(11):2762–2769. doi: 10.1002/jbmr.499 21887701

[8] Palomo T Muszkat P Weiler FG Dreyer P Brandão CMA Silva BC . Update on trabecular bone score. Archives of Endocrinology and Metabolism. (2022) 66(5):694–706. doi: 10.20945/2359-3997000000559 36382759 PMC10118821

[9] Boutroy S Bouxsein ML Munoz F Delmas PD . In vivo assessment of trabecular microarchitecture by HR-pQCT. Journal of Clinical Endocrinology & Metabolism. (2005) 90:6508–6515. doi: 10.1210/jc.2005-1258 16189253

[10] Nilsson M Sundh D Mellström D Lorentzon M . Type 2 diabetes is associated with impaired cortical microstructure. Journal of Bone and Mineral Research. (2017) 32:1641–1650. doi: 10.1002/jbmr.3057 27676223

[11] Rosen CJ Bouxsein ML . Mechanisms of disease: is osteoporosis the obesity of bone?. Nature Clinical Practice. Rheumatology. (2006) 2(1):35–43. doi: 10.1038/ncprheum0070 16932650

[12] Cao JJ . Effects of obesity on bone metabolism. Journal of Orthopaedic Surgery and Research. (2011) 6:30. doi: 10.1186/1749-799X-6-30 21676245 PMC3141563

[13] Wauquier F Leotoing L Coxam V Guicheux J Wittrant Y . Oxidative stress in bone remodelling and disease. Trends in Molecular Medicine. (2009) 15(10):468–477. doi: 10.1016/j.molmed.2009.08.004 19811952

[14] Wortsman J Matsuoka LY Chen TC Lu Z Holick MF . Decreased bioavailability of vitamin D in obesity. The American Journal of Clinical Nutrition. (2000) 72:690–693. doi: 10.1093/ajcn/72.3.690 10966885

[15] Yu EW Bouxsein ML Roy AE Baldwin C Cange A Neer RM . Bone loss after bariatric surgery. Journal of Bone and Mineral Research. (2014) 29:542–550. doi: 10.1002/jbmr.2226 23929784 PMC3918250

[16] Goldner WS Stoner JA Lyden E Thompson J Taylor K Larson L . Finding the optimal dose of vitamin D following Roux-en-Y gastric bypass: a prospective, randomized pilot clinical trial. Obesity Surgery. (2009) 19(2):173–179. doi: 10.1007/s11695-008-9680-y 18795378

[17] Drincic AT Armas LAG Van Diest EE Heaney RP . Volumetric dilution explanation for low vitamin D in obesity. Obesity. (2012) 20:1444–1448. doi: 10.1038/oby.2011.404 22262154

[18] Frost HM . Bone’s mechanostat: a 2003 update. The Anatomical Record. Part A, Discoveries in Molecular, Cellular, and Evolutionary Biology. (2003) 275:1081–1101. doi: 10.1002/ar.a.10119 14613308

[19] Turner CH . Three rules for bone adaptation to mechanical stimuli. Bone. (1998) 23:399–407. doi: 10.1016/S8756-3282(98)00118-5 9823445

[20] Bonewald LF . The amazing osteocyte. Journal of Bone and Mineral Research. (2011) 26:229–238. doi: 10.1002/jbmr.320 21254230 PMC3179345

[21] Klein-Nulend J Bakker AD Bacabac RG Vatsa A Weinbaum S . Mechanosensation and transduction in osteocytes. Bone. (2013) 54:182–190. doi: 10.1016/j.bone.2012.10.013 23085083

[22] Robling AG Turner CH . Mechanical signaling for bone modeling and remodeling. Critical Reviews in Eukaryotic Gene Expression. (2009) 19:319–338. doi: 10.1615/CritRevEukarGeneExpr.v19.i4.50 19817708 PMC3743123

[23] Robling AG Niziolek PJ Baldridge LA Condon KW Allen MR Alam I . Mechanical stimulation of bone in vivo reduces osteocyte expression of sclerostin. The Journal of Biological Chemistry. (2008) 283:5866–5875. doi: 10.1074/jbc.M705092200 18089564

[24] Sun W Chi S Li Y Ling S Tan Y Xu Y . The mechanosensitive Piezo1 channel is required for bone formation. eLife. (2019) 8:e47454. doi: 10.7554/eLife.47454 31290742 PMC6685704

[25] Ma W Liu Y Feng Z Liu X Jia G Geng B Xia Y . The saturation effect of body mass index on bone mineral density for people over 50 years old: a cross-sectional study of the US population. Frontiers in Nutrition. (2021) 8:763677. doi: 10.3389/fnut.2021.763677 34722617 PMC8554069

[26] Ferron M Wei J Yoshizawa T Del Fattore A DePinho RA Teti A . Insulin signaling in osteoblasts integrates bone remodeling and energy metabolism. Cell. (2010) 142:296–308. doi: 10.1016/j.cell.2010.06.003 20655470 PMC2910411

[27] Simpson ER . Aromatization of androgens in women: current concepts and findings. Fertility and Sterility. (2002) 77(Suppl 4):S6–S10. doi: 10.1016/s0015-0282(02)02984-9 12007896

[28] Gordeladze JO Drevon CA Syversen U Reseland JE . Leptin stimulates human osteoblastic cell proliferation, de novo collagen synthesis, and mineralization: Impact on differentiation markers, apoptosis, and osteoclastic signaling. Journal of Cellular Biochemistry. (2002) 85(4):825–836. doi: 10.1002/jcb.10156 11968022

[29] Takeda S Elefteriou F Levasseur R Liu X Zhao L Parker KL . Leptin regulates bone formation via the sympathetic nervous system. Cell. (2002) 111:305–317. doi: 10.1016/S0092-8674(02)01049-8 12419242

[30] Oshima K Nampei A Matsuda M Iwaki M Fukuhara A Hashimoto J . Adiponectin increases bone mass by suppressing osteoclasts. Biochemical and Biophysical Research Communications. (2005) 331:520–526. doi: 10.1016/j.bbrc.2005.03.210 15850790

[31] Luo XH Guo LJ Xie H Yuan LQ Wu XP Zhou HD . Adiponectin stimulates RANKL and inhibits osteoprotegerin. Journal of Bone and Mineral Research. (2006) 21:1648–1656. doi: 10.1359/jbmr.060707 16995820

[32] Ishii S Cauley JA Crandall CJ . Inflammation, adiposity, and bone in older adults: the Health ABC Study. Journal of Clinical Endocrinology & Metabolism. (2014) 99:2725–2733. doi: 10.1210/jc.2013-3469 24476075

[33] Boyle WJ Simonet WS Lacey DL . Osteoclast differentiation and activation. Nature. (2003) 423:337–342. doi: 10.1038/nature01658 12748652

[34] Domazetovic V Marcucci G Iantomasi T Brandi ML Vincenzini MT . Oxidative stress in bone remodeling: role of antioxidants. Clinical Cases in Mineral and Bone Metabolism. (2017) 14:209–216. doi: 10.11138/ccmbm/2017.14.1.209 29263736 PMC5726212

[35] Almeida M Han L Martin-Millan M O’Brien CA Manolagas SC . Oxidative stress antagonizes Wnt signaling. The Journal of Biological Chemistry. (2007) 282:27298–27305. doi: 10.1074/jbc.M702811200 17623658

[36] Saito M Marumo K . Collagen cross-links as a determinant of bone quality. Osteoporosis International. (2010) 21:195–214. doi: 10.1007/s00198-009-1066-z 19760059

[37] Cawthorn WP Scheller EL Learman BS Parlee SD Simon BR Mori H . Bone marrow adipose tissue as an endocrine organ. Cell Metabolism. (2014) 20:1046–1061. doi: 10.1016/j.cmet.2014.06.003 24998914 PMC4126847

[38] Shu A Yin MT Stein E Cremers S Dworakowski E Ives R . Bone structure and turnover in type 2 diabetes mellitus. Osteoporosis International. (2012) 23(2):635–641. doi: 10.1007/s00198-011-1595-0 21424265 PMC3690650

[39] Farr JN Drake MT Amin S Melton LJ McCready LK Khosla S . Bone microarchitecture and strength in type 2 diabetes mellitus. Journal of Bone and Mineral Research. (2014) 29:787–795. doi: 10.1002/jbmr.2106 24123088 PMC3961509

[40] Seale P Bjork B Yang W Kajimura S Chin S Kuang S . PRDM16 controls a brown fat-skeletal muscle switch. Nature. (2008) 454:961–967. doi: 10.1038/nature07182 18719582 PMC2583329

[41] Earthman CP Beckman LM Masodkar K Sibley SD . Obesity and vitamin D deficiency. International Journal of Obesity. (2012) 36:387–396. doi: 10.1038/ijo.2011.119 21694701

[42] Holick MF Binkley NC Bischoff-Ferrari HA Gordon CM Hanley DA Heaney RP . Endocrine Society guideline on vitamin D deficiency. Journal of Clinical Endocrinology & Metabolism. (2011) 96:1911–1930. doi: 10.1210/jc.2011-0385 21646368

[43] Ross AC Manson JE Abrams SA Aloia JF Brannon PM Clinton SK . 2011 dietary reference intakes for calcium and vitamin D. Journal of Clinical Endocrinology & Metabolism. (2011) 96:53–58. doi: 10.1210/jc.2010-2704 21118827 PMC3046611

[44] Schafer AL Weaver CM Black DM Wheeler AL Chang H Szefc GV . Intestinal calcium absorption after gastric bypass surgery. Journal of Bone and Mineral Research. (2015) 30:1377–1385. doi: 10.1002/jbmr.2467 25640580 PMC4593653

[45] Bischoff-Ferrari HA Dawson-Hughes B Staehelin HB Orav JE Stuck AE Theiler R . Fall prevention with vitamin D supplementation. BMJ. (2009) 339:b3692. doi: 10.1136/bmj.b3692 19797342 PMC2755728

[46] Ilich JZ Kelly OJ Inglis JE . Osteosarcopenic obesity syndrome: what is it and how can it be identified and diagnosed?. Current Gerontology and Geriatrics Research. (2016) 2016:7325973. doi: 10.1155/2016/7325973 27667996 PMC5030469

[47] Donini LM Busetto L Bischoff S Cederholm T Ballesteros-Pomar MD Batsis JA . Sarcopenic obesity definition and diagnostic criteria. Nutrition Reviews. (2022) 80:1117–1130. doi: 10.1093/nutrit/nuab099 34879147 PMC8653947

[48] Lighthouse JK Carragher DM . Mitochondrial dysfunction in sarcopenia. Aging Research Reviews. (2023) 85:101837. doi: 10.1016/j.arr.2023.101837 36634871

[49] Zanker J Duque G . Osteosarcopenia: the Path Beyond Controversy. Current Osteoporosis Reports. (2020) 18(2):81–84. doi: 10.1007/s11914-020-00567-6 32130628

[50] Deutz NEP Bauer JM Barazzoni R Biolo G Boirie Y Bosy-Westphal A . Protein intake and exercise for muscle function in aging. Clinical Nutrition. (2014) 33:929–936. doi: 10.1016/j.clnu.2014.04.007 24814383 PMC4208946

[51] Movassagh EZ Vatanparast H . Dietary patterns and bone health. Advances in Nutrition. (2017) 8:1–16. doi: 10.3945/an.116.013326 28096123 PMC5227978

[52] Weiss EP Jordan RC Frese EM Albert SG Villareal DT . Effects of Weight Loss on Lean Mass, Strength, Bone, and Aerobic Capacity. Medicine & Science in Sports & Exercise. (2017) 49(1):206–217. doi: 10.1249/MSS.0000000000001074 27580151 PMC5161655

[53] Howe TE Rochester L Neil F Skelton DA Ballinger C . Exercise for improving balance in older people. Cochrane Database of Systematic Reviews. (2011) 2011(11):CD004963. doi: 10.1002/14651858.CD004963.pub3 22071817 PMC11493176

[54] Liao CD Wu YT Tsauo JY Chen PR Tu YK Chen HC . Protein supplementation plus resistance training. Nutrients. (2020) 12(8):2422. doi: 10.3390/nu12082422 32806718 PMC7468926

[55] Neer RM Arnaud CD Zanchetta JR Prince R Gaich GA Reginster JY . PTH (1-34) and fracture reduction. The New England Journal of Medicine. (2001) 344:1434–1441. doi: 10.1056/NEJM200105103441904 11346808

[56] McClung MR Grauer A Boonen S Bolognese MA Brown JP Diez-Perez A . Romosozumab in low bone density. The New England Journal of Medicine. (2014) 370:412–420. doi: 10.1056/NEJMoa1305224 24382002

[57] Bhasin S Krishnan V Storer TW Steiner M Dobs AS . Androgens and Selective Androgen Receptor Modulators to Treat Functional Limitations Associated With Aging and Chronic Disease. The Journals of Gerontology. Series A, Biological Sciences and Medical Sciences. (2023) 78(Suppl 1):25–31. doi: 10.1093/gerona/glad027 37325955 PMC10272983

[58] Suh J Lee YS . Myostatin Inhibitors: Panacea or Predicament for Musculoskeletal Disorders?. Journal of Bone Metabolism. (2020) 27(3):151–165. doi: 10.11005/jbm.2020.27.3.151 32911580 PMC7571243

[59] Bolotin HH . DXA in vivo BMD methodology: an erroneous and misleading research and clinical gauge of bone mineral status, bone fragility, and bone remodelling. Bone. (2007) 41(1):138–154. doi: 10.1016/j.bone.2007.02.022 17481978

[60] Tothill P Avenell A . Errors in dual-energy X-ray absorptiometry of the lumbar spine owing to fat distribution and soft tissue thickness during weight change. The British Journal of Radiology. (1994) 67(793):71–75. doi: 10.1259/0007-1285-67-793-71 8298878

[61] Walsh JS Vilaca T . Obesity, Type 2 Diabetes and Bone in Adults. Calcified Tissue International. (2017) 100(5):528–535. doi: 10.1007/s00223-016-0229-0 28280846 PMC5394147

[62] Vilaca T Evans A Gossiel F Paggiosi M Eastell R Walsh JS . Fat, adipokines, bone structure and bone regulatory factors associations in obesity. European Journal of Endocrinology. (2022) 187(6):743–750. doi: 10.1530/EJE-22-0530 36173650 PMC9641785

[63] Harvey NC Glüer CC Binkley N McCloskey EV Brandi ML Cooper C . Trabecular bone score as a complementary approach for osteoporosis evaluation. Bone. (2015) 78:216–224. doi: 10.1016/j.bone.2015.05.016 25988660 PMC4538791

[64] Pothuaud L Barthe N Krieg MA Mehsen N Carceller P Hans D . Evaluation of the potential use of trabecular bone score to complement bone mineral density in the diagnosis of osteoporosis: a preliminary spine BMD-matched, case-control study. Journal of Clinical Densitometry. (2009) 12(2):170–176. doi: 10.1016/j.jocd.2008.11.006 19181553

[65] Chuang TL Chuang MH Wang YF Koo M . Comparison of Trabecular Bone Score-Adjusted Fracture Risk Assessment (TBS-FRAX) and FRAX Tools for Identification of High Fracture Risk among Taiwanese Adults Aged 50 to 90 Years with or without Prediabetes and Diabetes. Medicina. (2022) 58(12):1766. doi: 10.3390/medicina58121766 36556968 PMC9787568

[66] McCloskey EV Odén A Harvey NC Leslie WD Hans D Johansson H . A Meta-Analysis of Trabecular Bone Score in Fracture Risk Prediction and Its Relationship to FRAX. Journal of Bone and Mineral Research. (2016) 31(5):940–948. doi: 10.1002/jbmr.2734 26498132

[67] Shanbhogue VV Hansen S Frost M Jørgensen NR Hermann AP Henriksen JE Brixen K . Bone Geometry, Volumetric Density, Microarchitecture, and Estimated Bone Strength Assessed by HR-pQCT in Adult Patients With Type 1 Diabetes Mellitus. Journal of Bone and Mineral Research. (2015) 30(12):2188–2199. doi: 10.1002/jbmr.2573 26096924

[68] Whittier DE Boyd SK Burghardt AJ Paccou J Ghasem-Zadeh A Chapurlat R Engelke K Bouxsein ML . Guidelines for the assessment of bone density and microarchitecture in vivo using high-resolution peripheral quantitative computed tomography. Osteoporosis International. (2020) 31(9):1607–1627. doi: 10.1007/s00198-020-05438-5 32458029 PMC7429313

[69] Schwartz AV Vittinghoff E Bauer DC Hillier TA Strotmeyer ES Ensrud KE . T2DM, BMD, and fracture risk in older adults. Journal of the American Geriatrics Society. (2011) 59:2031–2038. doi: 10.1001/jama.2011.715 21632482 PMC3287389

[70] Giangregorio LM Leslie WD Lix LM Johansson H Oden A McCloskey E Kanis JA . FRAX underestimates fracture risk in patients with diabetes. Journal of Bone and Mineral Research. (2012) 27(2):301–308. doi: 10.1002/jbmr.556 22052532

[71] Chen W Mao M Fang J Xie Y Rui Y . Fracture risk assessment in diabetes mellitus. Frontiers in Endocrinology. (2022) 13:961761. doi: 10.3389/fendo.2022.961761 36120431 PMC9479173

[72] Leslie WD Johansson H McCloskey EV Harvey NC Kanis JA Hans D . Comparison of Methods for Improving Fracture Risk Assessment in Diabetes: The Manitoba BMD Registry. Journal of Bone and Mineral Research. (2018) 33(11):1923–1930. doi: 10.1002/jbmr.3538 29953670 PMC6193547

[73] Cairoli E Grassi G Gaudio A Palermo A Vescini F Falchetti A . Validation of the clinical consensus recommendations on the management of fracture risk in postmenopausal women with type 2 diabetes. Nutrition, Metabolism and Cardiovascular Diseases. (2023) 33(1):158–167. doi: 10.1016/j.numecd.2022.10.004 36404237

[74] Al-Daghri NM Sabico S Al-Saleh Y Sulimani R Aljohani NJ Sheshah E . Application of FRAX in Saudi Arabia: intervention thresholds. Archives of Osteoporosis. (2021) 16:166. doi: 10.1007/s11657-021-01024-2 34739604

[75] Lalmohamed A Welsing PM Lems WF Jacobs JW Kanis JA Johansson H . Calibration of FRAX® 3.1 to the Dutch population with data on the epidemiology of hip fractures. Osteoporosis International. (2012) 23(3):861–869. doi: 10.1007/s00198-011-1852-2 22120910 PMC3277691

[76] Tebé Cordomí C Del Río LM Di Gregorio S Casas L Estrada MD Kotzeva A Espallargues M . Validation of the FRAX predictive model for major osteoporotic fracture in a historical cohort of Spanish women. Journal of Clinical Densitometry. (2013) 16(2):231–237. doi: 10.1016/j.jocd.2012.05.007 22748778

[77] Cosman F de Beur SJ LeBoff MS Lewiecki EM Tanner B Randall S . Clinician’s guide to prevention and treatment of osteoporosis. Osteoporosis International. (2014) 25:2359–2381. doi: 10.1007/s00198-014-2794-2 25182228 PMC4176573

[78] Kanis JA Borgstrom F De Laet C Johansson H Johnell O Jonsson B . Assessment of fracture risk. Osteoporosis International. (2005) 16(6):581–589. doi: 10.1007/s00198-004-1780-5 15616758

[79] Center JR Bliuc D Nguyen TV Eisman JA . Risk of subsequent fracture after low-trauma fracture. Journal of the American Medical Association. (2007) 297(4):387–394. doi: 10.1001/jama.297.4.387 17244835

[80] Wu Y Chao J Bao M Zhang N . Predictive value of machine learning on fracture risk in osteoporosis: a systematic review and meta-analysis. BMJ Open. (2023) 13(12):e071430. doi: 10.1136/bmjopen-2022-071430 38070927 PMC10728980

[81] Yoo TK Kim SK Kim DW Choi JY Lee WH Oh E . Osteoporosis risk prediction for bone mineral density assessment of postmenopausal women using machine learning. Yonsei Medical Journal. (2013) 54(6):1321–1330. doi: 10.3349/ymj.2013.54.6.1321 24142634 PMC3809875

[82] Zebaze R Ghasem-Zadeh A Mbala A Seeman E . Deep learning analysis of HR-pQCT improves fracture discrimination. Bone. (2021) 153:116125. doi: 10.1016/j.bone.2021.116125 34280582

[83] Wang D Cao H Hua W Gao L Yuan Y Zhou X Zeng Z . Mesenchymal Stem Cell-Derived Extracellular Vesicles for Bone Defect Repair. Membranes. (2022) 12(7):716. doi: 10.3390/membranes12070716 35877919 PMC9315966

[84] Zhu Y Li J Chen X . PKM2-mediated metabolic reprogramming in osteogenic differentiation under high glucose inflammatory conditions. Stem Cell Research & Therapy. (2025) 16:186. doi: 10.1186/s13287-025-04301-w 40251642 PMC12008901

[85] Shigehara K Izumi K Kadono Y Mizokami A . Testosterone and bone health in men: narrative review. Journal of Clinical Medicine. (2021) 10(3):530. doi: 10.3390/jcm10030530 33540526 PMC7867125

[86] Bhasin S Jasuja R . Selective androgen receptor modulators as function-promoting therapies. Current Opinion in Clinical Nutrition and Metabolic Care. (2009) 12(3):232–240. doi: 10.1097/MCO.0b013e32832a3d79 19357508 PMC2907129

[87] Becker C Lord SR Studenski SA Warden SJ Fielding RA Recknor CP . Myostatin antibody (LY2495655) in older weak fallers: randomized phase 2 trial. The Lancet Diabetes & Endocrinology. (2015) 3(12):948–957. doi: 10.1016/S2213-8587(15)00298-3 26516121

